# Immunotherapy in Early-Stage Triple-Negative Breast Cancer: Where Are We Now and Where Are We Headed?

**DOI:** 10.1007/s11864-023-01087-y

**Published:** 2023-05-24

**Authors:** Julia Dixon-Douglas, Sherene Loi

**Affiliations:** 1 Division of Research, Peter MacCallum Cancer Centre, 305 Grattan St, Melbourne, Australia; 2 The Sir Peter MacCallum Department of Medical Oncology, University of Melbourne, Parkville, Australia

**Keywords:** Immune checkpoint inhibitors, Early-stage triple-negative breast cancer, Neoadjuvant therapy, PD-1, Tumour-infiltrating lymphocytes

## Abstract

Recently, the addition of PD-1 pathway targeting immune checkpoint inhibitors (ICI) to standard neoadjuvant chemotherapy for early-stage triple-negative breast cancer (TNBC) has been shown to improve rates of pathological complete response (pCR), as well as event-free survival regardless of attainment of pCR. Recurrent TNBC remains a devastating diagnosis and thus novel treatments that improve chance of cure in early-stage TNBC should be promptly integrated into standard of care paradigms. However, approximately 50% of patients with early TNBC will experience pCR with chemotherapy alone, and the addition of ICI carries the risk of sometimes permanent immune-related toxicities. This raises the critical question whether all early-stage TNBC patients should receive ICI in combination with neoadjuvant chemotherapy. As yet, there is no predictive biomarker to select patients most likely to benefit from ICI; however, it would seem that at least all node positive patients should receive an ICI with their neoadjuvant chemotherapy, on the basis of high clinical risk and potential to increase their pCR rate and ultimately the chance of cure. It is plausible that some lower-risk (stage I/II) TNBC demonstrating strong pre-existing immune activation (high tumor-infiltrating lymphocytes (TILs) and/or PD-L1 expression) may be successfully treated with ICI in combination with less cytotoxic chemotherapy, and this requires further evaluation in clinical trials. The contribution of the adjuvant phase of ICI on clinical benefit is unclear even in patients who do not achieve a pCR and long-term data from ongoing studies without adjuvant ICI component may help inform us on an appropriate strategy in the short term. Similarly, the potential benefit of other adjuvant therapies in patients with poor response to neoadjuvant ICI with chemotherapy, including capecitabine and olaparib with or without ICI, is also unknown, but is rational on the basis of administering a non-cross-resistant anti-tumour agent. In conclusion, the addition of neoadjuvant ICI to chemotherapy significantly improves both the quality and quantity of the anti-tumour T cell response, suggesting that improvements in recurrence-free survival occur through better immune protection from cancer. In the future, development of ICI agents that target tumour-specific T cells may favourably alter the toxicity profile, improving the risk–benefit ratio for survivors.

## Introduction

Triple-negative breast cancer (TNBC) is an aggressive histological subtype with high risk of distant metastatic recurrence and death, even when diagnosed in the early stage. For many years, standard (neo)adjuvant systemic therapy for early TNBC has been limited to anthracycline- and taxane-based chemotherapy. Although breast cancer is not traditionally considered an immunogenic tumour, the presence of tumour-infiltrating lymphocytes (TILs) is recognised as a good prognostic factor in early TNBC [1, 2•, 3], indicating the importance of immune surveillance in control of this disease. Immune checkpoint inhibitors (ICI) targeting programmed death-1 (PD-1) and its ligand (PD-L1) now have an established role in the first-line treatment of advanced TNBC and have recently been approved for use in early-stage TNBC. Several randomised phase II and III clinical trials have now shown improved pCR and event-free survival (EFS) with the addition of ICI to neoadjuvant chemotherapy in early TNBC, bringing us to a new standard of care for early TNBC. Importantly, ICI also significantly improves event-free survival even in patients who do not experience pCR [4•]. However, the risk of recurrence in patients with residual disease remains clinically significant, and outcomes remain dismal in the small proportion of patients who demonstrate primary resistance with high burden of residual disease despite maximal neoadjuvant chemo-ICI. On the other hand, financial cost and risk of potentially permanent immune-related toxicities is not insignificant, and some patients can be cured with chemotherapy alone. At present, important questions to consider are how to select patients for therapy and what is the optimum combination, sequence, and duration of therapy to maximise potential benefit from ICI. As the use of chemo-ICI becomes a standard of care in early TNBC, addressing primary resistance (high burden of residual disease) and secondary resistance (recurrence after curative-intent chemo-ICI) are major research priorities.

### Current treatment landscape

#### Efficacy of immune checkpoint inhibitors in early stage TNBC

Results are available for three randomised phase III and three randomised phase II clinical trials evaluating the addition of ICI to neoadjuvant chemotherapy in early TNBC. These are summarised in Table 1. KEYNOTE-522 can be considered the seminal trial demonstrating a statistically and clinically significant improvement in both pCR and EFS with the addition of pembrolizumab to neoadjuvant chemotherapy, consisting of carboplatin and paclitaxel followed by 3-weekly doxorubicin and cyclophosphamide (AC) [5••, 6••]. The phase III IMpassion031 trial also demonstrated an improvement in pCR with the addition of atezolizumab to neoadjuvant chemotherapy [7••], although EFS data is pending. There have been some inconsistencies amongst the results of other trials. Most notably no significant pCR benefit for atezolizumab was seen in the phase III NeoTripaPDL1 trial or the phase II GeparNuevo trial [8••, 9•]. The GeparNuevo trial featured a unique, 2-week lead-in durvalumab monotherapy or placebo window period for the first 117 patients treated (later amended due to concerns regarding delay to chemotherapy). When the analysis was restricted to this pre-defined window subgroup, a significant pCR benefit was in fact seen for the durvalumab arm (pCR 61.0% for durvalumab arm versus 41.4% for placebo arm, OR 2.22 95% CI 1.06–4.64; *p* = 0.035). Furthermore, despite the lack of significant pCR improvement, a statistically significant 3-year distant disease-free survival benefit was seen in the durvalumab arm in the overall population [10••], further supporting the long-term benefit of the addition of ICI in early TNBC.

**Table 1:** Select trials of neoadjuvant immune checkpoint inhibitors + chemotherapy in early-stage TNBC

Trial	Stage	N+ (%)	ICI	Chemotherapy Backbone	Adjuvant Therapy	Results
Randomised Phase III						
**KEYNOTE-522** *N *= 1174	cT1N1-2, cT2-4N0-2	51.7	Pembro	Carbo + paclitaxel, then AC (non-dd)	Pembro, 1 year total *No cape allowed*	pCR in ITT 64.8% vs 51.2%, *p* <0.001 3-year EFS 84.5% vs 76.8%, HR 0.63, *p* < 0.001
**IMpassion031** *N *= 333	cT2-4N0-3	34	Atezo	Nab-paclitaxel, then ddAC	Atezo, 1 year total *Cape allowed for RD*	pCR in ITT 58% vs 41%, *p *= 0.0044 pCR in PD-L1+ 69% vs 49%, *p *= 0.021 EFS immature
**NeoTRIPaPDL1** *N *= 280	cT1N1-3, cT2-4dN0-3	88	Atezo	Carbo + nab-paclitaxel	AC/EC/FEC	pCR 48.6% vs 44.4%, *p *= 0.48 EFS immature
Randomised Phase II						
**GeparNuevo** *N *= 174	cT2-T4dN0-3	31.4	Durva	Window: 2 weeks durva alone pre-chemo Nab-paclitaxel, then ddAC	No adjuvant therapy	pCR 53.4% vs 44.2%, OR 1.45 3-year DDFS 91.4% vs 79.5% , *p *= 0.0148 3-year OS 95.1% vs 83.1%, HR 0.26, *p *= 0.007
**I-SPY2** *N *= 69	cT2-4dN0-3	43	Pembro	Paclitaxel, then AC (dd or non-dd)	Clinicians’ discretion	pCR 60% vs 22% in TNBC cohort
**NCI10013** *N *= 67	cT2-4Nany	NA	Atezo	Carbo + paclitaxel	ddAC	pCR: 55.6% vs 18.8%, p-value 0.018
Single-arm Phase II						
**NeoPACT** *N *=117	Stage I-III	39	Pembro	Carbo + docetaxel	Clinician’s discretion	pCR: 60% overall 2-year EFS 89%
**CHARIOT** *N *= 34	Stage III ≥ 15mm RD after AC x 4 47% N+	-	Ipi + Nivo	Paclitaxel (AC received prior to randomization)	Nivo	pCR 24.2% 12-month EFS 85%
**Neo-N**	cT1cN1, cT2-4N0-1	-	Nivo	Window: 2 weeks nivo alone pre- or post-chemo Carbo + paclitaxel	Clinicians’ discretion	ongoing
Combination with PARP-i						
**I-SPY2** *N *= 73 (21 TNBC, 52 HR-positive, HER2 negative)	Stage II-III	29	Durva + olaparib	Paclitaxel, then AC (dd or non-dd)	No adjuvant therapy	pCR 47% vs 27% in TNBC cohort pCR 14% vs 28% in ER+ cohort

*TNBC* triple negative breast cancer, *HR-positive* hormone receptor positive, *ICI* immune checkpoint inhibitor, *N+* node-positive, *pembro* pembrolizumab, *atezo* atezolizumab, *durva* durvalumab, *nivo* nivolumab, *ipi* ipilimumab, *carbo* carboplatin, *AC* doxorubicin and cyclophosphamide, *EC* epirubicin and cyclophosphamide, *F* 5-fluorouracil, *cape* capecitabine, *PARP-i* PARP inhibitor, *dd* dose-dense (2-weekly), *non-dd* non-dose-dense (3-weekly), *pCR* pathological complete response, *EFS* event-free survival, *DDFS* distant disease-free survival, *OS* overall survival, *ITT* intent to treat, *RD* residual disease, *PD-L1* programmed death ligand-1, *HR* hazard ratio

#### Is there an optimal chemotherapy backbone?

There are some notable differences between the patient populations and chemotherapy backbones between the trials outlined above. NeoTrip-aPDL-1 included patients with N3 disease (15%), whilst these patients were excluded from KEYNOTE-522 and constituted only 5% of patients in IMpassion031. Despite having a higher risk patient population with 88% node positive patients in NeoTripaPDL1, only carboplatin and nab-paclitaxel were administered in the neoadjuvant phase, and AC was given post-operatively, possibly explaining the relatively low pCR rate in both arms of this study (Table 1). IMpassion031, I-SPY2 and GeparNuevo all yielded positive results with anthracycline, cyclophosphamide and paclitaxel, without the addition of carboplatin. These trials all utilised dose-dense (2-weekly) AC, in contrast to 3-weekly AC in KEYNOTE-522. These differences in chemotherapy backbone add to existing uncertainty regarding the additional benefit carboplatin in early TNBC. Whilst the BrighTNess trial demonstrated EFS benefit for the addition of carboplatin to paclitaxel followed by 3-weekly AC and GeparSixto showed significantly improved pCR and disease-free survival with the addition of carboplatin to paclitaxel and doxorubicin, the addition of carboplatin to weekly paclitaxel followed by dose-dense AC improved pCR but not long-term outcomes in CALGB 40603 [11–13]. As the largest phase III trial to date, and the only trial to demonstrate statistically significant benefit in both pCR and EFS with the addition of ICI, the regimen utilised in KEYNOTE-522 may be considered the current standard of care. This is further supported by US Food and Drug Administration (FDA) approval of pembrolizumab for early-stage TNBC in light of the KEYNOTE-522 results. However, whether the chemotherapy backbone can be further refined by reducing or individually tailoring the extent of cytotoxic therapy requires further evaluation in future clinical trials.

#### Adjuvant therapy considerations

Whether adjuvant ICI is necessary after neoadjuvant therapy is unclear. Both KEYNOTE-522 and IMpassion031 included post-operative ICI to complete a total of one year of therapy for responders and non-responders alike. In contrast, GeparNuevo showed an EFS benefit for neoadjuvant ICI with no adjuvant therapy. This benefit was seen across those who did and did not experience pCR, suggesting that the EFS benefit of ICI can be driven by neoadjuvant administration even in the case of residual disease. The EFS benefit in KEYNOTE-522 was again seen in patients with pCR and non-pCR, with the greatest improvement in EFS seen in those with moderate residual burden of disease (residual cancer burden [RCB] class II)[4•]. Despite these improvements, prognosis remains dismal for patients with RCB-III (3-year EFS 26.2% and 34.6% with and without pembrolizumab in KEYNOTE-522, respectively) and represents an area of unmet need. The question of optimum adjuvant therapy post ICI remains unanswered, and the difference in EFS according to level of RCB in KEYNOTE-522 indicates that a tailored approach to adjuvant therapy could be evaluated in future clinical trials, with a particular focus on novel and combination therapies for RCB-III. One such trial, TROPION-Breast03 (NCT05629585), is evaluating the antibody–drug conjugate datopotamab deruxtecan, with or without durvalumab, as adjuvant therapy in patients with residual disease following neoadjuvant systemic therapy, including both patients who did and did not receive an ICI as part of their neoadjuvant regimen. The important contrary question of whether patients who do experience pCR can be spared adjuvant therapy is being evaluated in the Optimice-pCR trial (NCT05812807), randomising patients who received neoadjuvant therapy including at least 6 cycles of pembrolizumab to observation versus ongoing pembrolizumab.

#### Immune-mediated toxicity: a serious issue

Whilst generally well-tolerated, ICI have the potential to cause serious, sometimes permanent life-threatening or life-altering immune-related adverse events (irAE). The most common irAE are rash, fatigue and thyroid dysfunction. Although more serious irAE, including permanent endocrinopathies such as adrenalitis, hypophysitis and type 1 diabetes mellitus are generally rare, the unpredictable and idiosyncratic nature of irAE presents a challenge, particularly in the early-stage setting, where some patients will be cured with standard chemotherapy alone. Recent data suggests that there may be an increased incidence of some irAE in young women [14, 15•]. In KEYNOTE-522, any-grade irAE occurred in 33.5% of patients receiving pembrolizumab (compared to 11.3% in the placebo group), including hypothyroidism in 15.1%, adrenal insufficiency in 2.3% and hypophysitis in 1.8%. Adrenal insufficiency (either as a result of hypophysitis or primary adrenal insufficiency) was reported in 8.7% of patients treated with pembrolizumab in I-SPY2, higher than previously reported for anti-PD-1 therapy [16•]. Recently, a preclinical study has reported that ICIs mediate ovarian inflammation and reduce oocyte reserves in a murine model [17•]. If these findings are confirmed in humans, this could have implications for all cancer patients of reproductive age receiving these treatments in the curative setting. This emphasises the essential need to integrate fertility research into future clinical trials. These life-altering toxicities highlight the need to refine patient selection for neoadjuvant ICI and to confirm the role and duration of ongoing adjuvant ICI in the post-neoadjuvant setting, particularly for those who experience pCR or near-pCR.

#### Patient selection in an era of ICI

As yet, no single predictive biomarker has been identified to select patients for neoadjuvant ICI for early-stage TNBC. In advanced TNBC, PD-L1 expression is required to benefit significantly from ICI. In early TNBC, PD-L1 has consistently been shown to be prognostic but not predictive, with improved pCR rates seen in PD-L1 positive tumours regardless of treatment with ICI or with chemotherapy alone across the KEYNOTE-522, IMpassion031 and GeparNuevo trials. Only the NeoTripaPDL1 trial showed an association between PD-L1 status and response [9•, 18•]. One possible explanation is that PD-L1 expression may be a more important determinant of response to ICI in patients with higher burden early-stage disease due to greater immune-exhaustion or suppression and a host immune milieu more similar to metastatic or widespread disease. Biomarker analyses of GeparNuevo and NeoTripaPDL1 have shown that TILs again are prognostic but not predictive of benefit from the addition of ICI to chemotherapy [8••, 18•]. This is keeping with the consistent observation that lower levels of TIL infiltrate are present in metastatic disease [19, 20•]. In the absence of a reliable biomarker, clinical risk currently remains the most pragmatic tool to select patients for the addition of ICI to neoadjuvant chemotherapy, and we recommend offering neoadjuvant ICI in combination to all node-positive patients. Other nuances with regard to biomarker development for better patient selection as well as the probably immune mechanisms of action of traditional chemotherapy are discussed below.

### Future therapeutic considerations and directions

**Fig. 1:**
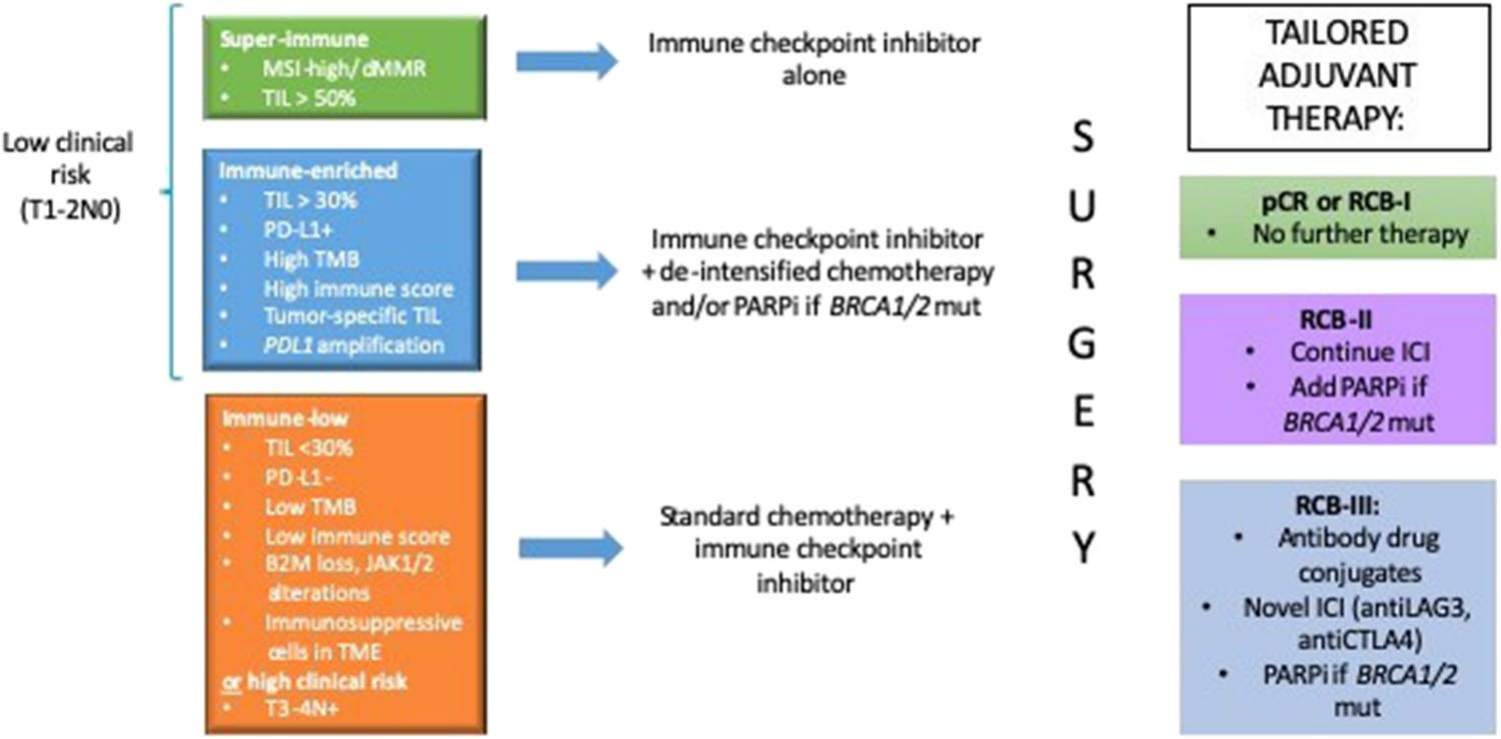
Proposed future strategies for tailoring ICI in early-stage TNBC. De-intensification of the cytotoxic chemotherapy backbone may be feasible for patients with high levels of pre-existing immune activation combined with lower clinical risk. Currently high level of tumour-infiltrating lymphocytes (TIL) and PD-L1 are markers of immune activation. Selection of patients for de-intensification could be improved with ongoing refinement of other biomarkers including tumour mutational burden (TMB), homologous recombination deficiency (HRD) such as *BRCA1/2* mutations, and use of immune gene signatures or scores. For patients with HRD, PARP inhibitor could also be incorporated to neoadjuvant immune checkpoint inhibition. Patients whose tumours lack these features have genomic features associated with immune resistance, such as B2M loss and JAK1/2 mutations, and those with high clinical risk should receive the current standard chemotherapy with ICI. The post-surgical phase provides the opportunity to escalate treatment with non-cross-resistant therapies designed to overcome resistance in patients with sub-optimal response. MSI, microsatellite instability; dMMR, mismatch repair deficiency; PD-L1, programmed death ligand-1; BRCA 1/2 mut, germline mutations in BRCA 1/2; TME, tumour microenvironment.

Several important clinical questions need to be addressed to optimise use of ICI. Specifically, which patients may be cured with chemotherapy alone (ICI not required), which patients can be cured with ICI in combination with less intensive cytotoxic chemotherapy and which patients will need more therapy than the current standard chemo-ICI?. These questions broadly arise from the issue of heterogeneity in TNBC, necessitating a biomarker-directed approach to individually tailor therapy (Fig. 1).

#### Biomarker development

The limitation of TILs and PD-L1 as biomarkers in early-stage TNBC is outlined above. Several trials have incorporated exploratory analyses to identify other potential biomarkers**.** In GeparNuevo, an increase in intra-tumoural TILs on biopsy on cycle 1, day 15 compared to baseline was the strongest predictor of response in the durvalumab arm in multivariate analysis (OR 9.36, 95% CI 1.26–69.5, *p* = 0.029), whereas this did not predict response in the placebo arm [8••]. In NeoTripaPDL1, however, stromal TILs at cycle 2, day 1 predicted response regardless of treatment arm [18•]. NeoTripaPDL1 also included analysis of the spatial arrangement of PD-L1 expression relative to TILs using imaging mass cytometry and single-cell RNA sequencing, showing that the degree of spatial connectivity between immune cells and epithelial cells predicts response to immunotherapy more so that TILs alone [21].

Certain genomic features are associated with an immune-activated or immune-exhausted microenvironment, indicating a process of co-evolution between tumour and immune microenvironment. For example, *TP53* loss is associated with poor response to therapy and poor prognosis in TNBC and has been linked to reduced infiltration of cytotoxic T cells and suppression of immunity through down-regulation of the GMP-AMP signalling (cGAS) stimulator of interferon genes (STING) pathway [22]. In contrast, genomic alterations that result in defective DNA damage repair pathways, such as *BRCA1/2*, *PALB2* and others, are associated with upregulation of this pathway [23, 24]. Other genomic features such as mismatch repair deficiency (dMMR) resulting in microsatellite instability (MSI), and high tumour mutational burden (TMB), have been associated with improved response to ICI in several types of cancer, including some patients with TNBC [25]. Whilst the former is extremely uncommon in TNBC, high TMB (greater than 10 mutations per megabase [mut/Mb]) is seen in 3–5% of patients with breast cancer and is more common in TNBC and metastatic disease (approximately 10%) [26, 27, 28•] and has been evaluated as a potential biomarker to predict ICI response in both the advanced and early-stage TNBC setting [29, 30, 31•]. GeparNuevo has reported a TMB analysis, demonstrating that whilst median TMB was low (1.53 mut/Mb), the median TMB was significantly higher in patients who experienced pCR (1.87 mut/Mb vs 1.39 mut/Mb, *p* = 0.005)[31•]. There was a statistically significant increase in the odds ratio for pCR per mutation per megabase of TMB in both treatment arms. Several studies have retrospectively evaluated multi-gene assays, including the 27-gene Determa-IO and the 53-gene Imprint signatures, largely based on high expression of immune checkpoint genes including *CTLA4, CD274* (encoding PD-L1) and *PDCD1* (encoding PD-1) [32, 33••, 34]. These signatures are yet to be evaluated in prospective, randomised trials.

The identification of tumour antigen–specific intra-tumoural T cells is of high interest, as many of the immune cells present are considered “bystander”: recruited by cytokines to the tumour microenvironment (TME) but not specific to the tumour antigen [35]. Lately markers such as CD39 and CD103 have been proposed to distinguish tumour-specific CD8^+^PD1^+^ T cells from those that are bystander. The presence of other immune checkpoints such as LAG3 and TIM3, oligoclonal T cell receptor repertoires as well as production of CXCL13 may also assist in this regard [36•]. These markers may help us determine which patients do or do not have robust tumour-specific immune responses, as these seem to be the T cells that are amplified following ICI. Furthermore, these markers may also help us design better immune agents that target tumour-specific T cells and may therefore carry less risk of immune-related adverse events. The role of the bystander T cells is unclear, and their presence in large quantities seems to suggest that for many of the intratumoural T cells present, neither activation nor antigen is required. It is possible that they contribute and facilitate the anti-tumour response, particularly in the initial stages whilst the generation of tumour-specific T cells is ongoing. Future research will delineate their role, functional state, and if they can be harnessed for therapeutic response.

Biomarkers that predict resistance to ICI are also important to consider, particularly with respect to developing strategies to overcome resistance. Although these have not been a focus of clinical trials, both tumour intrinsic and extrinsic factors have been identified as putative markers of resistance. The presence of immunosuppressive cells such as tumour-associated macrophages (TAMs), myeloid derived suppressor cells (MDSCs) and T regulatory cells in the TME is associated with resistance and is likely to be at least partially driven by tumour intrinsic features that affect antigen-presentation or interferon signalling [37]. Impaired antigen presentation as result of loss of *B2M* (encoding beta-2-microglobulin) and *HLA-A* deletion is associated with an immune-desert TME in breast cancer [38, 39]. *B2M* loss has also been linked to ICI resistance in melanoma patients and in a murine model of TNBC [40, 41]. Loss-of-function alterations in the interferon-receptor associated Janus kinase 1 and 2 (*JAK1/2*) have similarly been associated with immune resistance [40, 42].

Biomarker analyses to date illustrate the importance of cell–cell interactions and dynamic changes in the TME as well as genomic features in determining response to ICI and indicate that a sophisticated approach to biomarker development will be required. At present, spatial analyses and single-cell profiling require significant expertise, time and investment, and whilst they will be instrumental for understanding immune biology, their utility in the clinic is less clear. The rapid development of artificial intelligence will also help to streamline biomarker analyses, as well as integrate multiple clinical, pathologic and genomic features, into predictive algorithms which may be clinically useful in the future, especially as multiple different immune-targeting agents are developed.

#### Chemotherapy de-intensification

The treatment of high-risk breast cancer patients has always involved cytotoxic chemotherapy. Yet, high levels of tumoural immune infiltrate, particularly higher levels of CD8^+^ T cells, also predict better responses to traditional chemotherapy (without ICI), including higher rates of pCR, implicating an immune mechanism of action. Chemotherapy likely also enhances tumour antigen creation and presentation. In our opinion, this indicates that the attainment of pCR is an immune response: a surrogate of effective tumour-specific immunity and the direct result of cytotoxic CD8^+^ T cell activity. Altogether, this suggests that intensive chemotherapy regimens could be shortened with the addition of ICI, particularly in the neoadjuvant phase where there is opportunity for subsequent escalation or de-escalation of systemic therapy post-surgery, according to response (Fig. 1). De-intensification of the chemotherapy component of neoadjuvant treatment is appealing on several levels. Firstly, this presents an opportunity to reduce short- and long-term toxicity (such as, cardiomyopathy, secondary haematological malignancies) for patients, as well as reducing the psychosocial burden of treatment. Secondly, shorter durations of chemotherapy could reduce demand on healthcare systems. Finally, there is a potential for the cytotoxic component of combination chemo-immunotherapy to abrogate long-term anti-tumour immunity, although this is not fully understood. There is evidence from the pre-immunotherapy era that the quality and quantity of circulating naïve T cells may never fully recover following adjuvant chemotherapy, and this appears to be greatest following combination anthracycline and taxane chemotherapy [43, 44]. Furthermore, reduced CD4 + naïve T cells and poor T cell receptor clonal diversity are associated with worse outcomes in metastatic breast cancer [45, 46]. The impact of chemotherapy-induced lymphopenia on clinical benefit from ICI when administered in either the early-stage setting or in case of distant recurrence is yet to be established in breast cancer. How treatment can be optimised to reduce the risk of immune-cell depletion and enhance response to ICI in breast cancer warrants further investigation and consideration in future clinical trial design.

The possibility of anthracycline-free regimens for certain patients with early-stage TNBC is already being explored. The randomised phase II NCI10013 trial showed a significant pCR benefit for the addition of atezolizumab to neoadjuvant carboplatin and paclitaxel. Whilst the pCR rate in the control arm was very low (18.8%), the pCR rate in the atezolizumab arm was comparable to that seen in other trials (55.6%). AC was given to all patients post-operatively in this trial, and the high pCR rates achieved with atezolizumab, carboplatin and paclitaxel alone raises the possibility of anthracycline-free regimens for a subset of patients. The single-arm phase II NeoPact trial evaluated 6 cycles of neoadjuvant pembrolizumab, carboplatin and docetaxel and achieved an overall pCR rate of 60% in the overall population and up to 78% patients with TILs greater than 30%[33••]. Preliminary survival data is also promising, with a 2-year EFS of 89%. The pCR in the node-positive population was 46% compared to 65% in the node-negative population. The Neo-N trial (ACTRN12619001308189) is evaluating a similar regimen of nivolumab with carboplatin and paclitaxel in patients with Stage IA-IIB TNBC. Currently, patient selection for these de-intensification trials is based on lower risk clinical stage. However, the identification of predictive biomarkers will ultimately be most helpful to refine patient selection for such an approach (Fig. 1).

#### Chemotherapy-free approaches

Recently, the non-randomised, window-of-opportunity BELLINI trial showed that responses can be achieved with ICI alone in some patients with early-stage TNBC [47•]. All patients had at least 5% TILs on baseline biopsy and received either nivolumab 240 mg 2-weekly (*n* = 15) or nivolumab 240 mg 2-weekly with one dose of ipilimumab 1 mg/kg (*n* = 15) for 4 weeks, before undergoing MRI, biopsy and proceeding to either surgery or chemotherapy. Although the primary endpoint of this trial was biological, a partial radiographic response was seen in 19% of patients receiving nivolumab and 27% of patients receiving combination ICI. Notably, all responders were patients with TILs of 40% or higher at baseline. Three patients went on to surgery directly after ICI (without neoadjuvant chemotherapy) and one of these patients (who received nivolumab monotherapy) experienced a pCR. Whilst this approach is clearly experimental, the results do confirm that a subset of patients with TNBC are sensitive to ICI alone and chemotherapy could be replaced in this context.

#### Overcoming primary and secondary resistance to PD-1 targeting agents

Whilst de-intensification of therapy may be feasible in patients who are sensitive to ICI, more effective therapies for the 5–6% of patients with poor or no response to standard neoadjuvant chemo-ICI are required [4•]. Biomarker analyses may shed light on mechanisms of resistance and lead to novel therapeutic strategies for tumours predicted to demonstrate resistance to therapy. Until the ability to predict for primary resistance is established, escalation of therapy for non-responders will be largely achieved by tailoring adjuvant therapy for residual disease post neoadjuvant chemo-ICI. Currently, there is no established standard of care for such patients. The current practice of adjuvant capecitabine, or olaparib in the case of patients harbouring *BRCA1/2* mutations, is of unknown benefit following neoadjuvant ICI. In KEYNOTE-522, all patients continued adjuvant pembrolizumab or placebo; adjuvant capecitabine was not allowed. In IMpassion-031, adjuvant capecitabine was allowed in addition to adjuvant atezolizumab according to investigator discretion. The dismal EFS outcomes seen in patients with RCB-III in KEYNOTE-522 highlights the need for clinical trials evaluating alternative strategies for high-risk residual disease, which might include additional chemotherapy, PARP-inhibitors, immunotherapy combinations, antibody drug conjugates, or other novel agents.

In the future, novel, synergistic, immunotherapeutic combinations in development may be employed in the neoadjuvant setting to improve pCR rates and limit the number of patients requiring “rescue” adjuvant therapy. DNA-damage repair inhibitors, such as PARP-inhibitors (PARPi), have the potential to enhance response to ICI through increased neo-antigen release, increased PD-L1 expression in the tumour microenvironment (TME) and increased immune-activating interferon signalling through upregulation of the cGAS-STING pathway [48]. This, as well as the immune-activated state seen in DNA-damage repair-deficient tumours, provides the biological rationale for such an approach. Early-phase studies, primarily in the metastatic setting, provide support for evaluating this approach further as a strategy in early-stage disease. The single-arm phase I/II TOPACIO and MEDIOLA trials have demonstrated safety and preliminary clinical activity of niraparib in combination with pembrolizumab and olaparib in combination with durvalumab, respectively, for treatment of advanced TNBC [49, 50]. The outcome of a randomised phase II trial evaluating olaparib alone or in combination with atezolizumab (NCT02849496) in metastatic homologous recombination deficient HER-2 negative breast cancer will provide further data regarding the true synergy of PARPi and ICI in clinical terms in the metastatic setting. One arm of the platform I-SPY2 trial treated 73 patients with HER-2 negative early-stage breast cancer (21 patients with TNBC and 52 patients with hormone-receptor positive breast cancer) with a combination of neoadjuvant durvalumab, olaparib and paclitaxel, followed by AC, prior to surgery, and demonstrated a pCR rate of 47% compared to 27% in the control arm (chemotherapy alone) in the TNBC cohort [51•]. This met the primary endpoint of ≥ 85% probability of superiority of the experimental arm, with manageable toxicity profile, warranting further investigation of ICI/PARPi ± chemotherapy combinations in the early-stage setting.

Dual immune-checkpoint blockade, which has demonstrated excellent pCR rates in melanoma, is another potential strategy which may improve pCR rates in certain patients. The single-arm, phase II CHARIOT trial investigated the addition of combination ipilimumab and nivolumab with weekly paclitaxel in patients with stage III TNBC with a suboptimal clinical response to neoadjuvant AC and achieved a pCR rate of 24.4% overall [52•], demonstrating feasibility of such an approach. Whilst agents targeting CTLA-4 have been associated with higher rates of immune adverse events, newer CTLA-4 agents have improved side effect profiles and may become more widely investigated.

As the use of neoadjuvant ICI becomes routine, understanding the benefit of ICI rechallenge in the setting of metastatic recurrence will be important. Should anti-PD-1 agents be continued in combination with other cytotoxic agents in the case of PD-L1 positive tumours? Will benefit from ongoing ICI depend on the disease-free interval? Should another checkpoint inhibitor be added? This will ultimately depend on identifying mechanisms of resistance to ICI, which are likely to be complex and heterogeneous. The role of B cells, particularly the importance of tertiary lymphoid structures, and other innate immune components such as natural killer cells, tumour-associated macrophages and dendritic cells, is another avenue of exploration to enhance anti-tumour immunity outside the scope of this review [53]. Translational research evaluating tumour and blood samples from patients at the time of metastatic recurrence after chemo-ICI will be required.

## Conclusion

The addition of ICI to standard neoadjuvant chemotherapy regimens has shown promising improvements, leading to a new standard of care in the treatment of early-stage triple-negative breast cancer. However, further work is required to maximise the potential benefit that can be derived from ICI in this disease. Currently, the optimal design and duration of the chemotherapy backbone is yet to be determined. De-intensification of chemotherapy is very likely feasible in certain patients with a high likelihood of response to ICI and identification of accurate biomarkers is critical. A combination of high levels of immune infiltrate, particularly CD8 + T cells and tumour-specific T cells, high TMB and early dynamic on-treatment changes will likely be helpful in this regard. The ideal adjuvant therapy post-neoadjuvant ICI — be it ongoing immunotherapy, chemotherapy or other agents — is unclear, and evaluation of tailored adjuvant therapy according to extent of residual disease will be required. Clinical trials evaluating novel strategies for patients with a high burden of residual disease post chemo-ICI are needed, with the long-term aim of developing the ability to understand and predict resistance in order to escalate neoadjuvant therapy upfront to achieve pCR for such patients. Improved understanding of the long-term impact of, and risk factors for, the development of serious immune-related adverse events is needed to mitigate potential toxicity associated with these agents. Development of immunotherapy agents that specifically target tumour-specific T cells could be helpful in abrogating these adverse events. Despite these limitations, the high risk of relapse and the paucity of effective therapies for advanced TNBC makes incremental improvements in event-free survival clinically significant, and chemo-ICI should be considered a standard of care for stage II–III TNBC.
